# Circulating Tumor Cells: From the Laboratory to the Cancer Clinic; A Closing Comment

**DOI:** 10.3390/cancers15030939

**Published:** 2023-02-02

**Authors:** Noyiyoshi Sawabata

**Affiliations:** Department of Thoracic and Cardio-Vascular Surgery, Nara Medical University School of Medicine, 840 Shijo-cho, Kashihara 634-8552, Japan; nsawabata@naramed-u.ac.jp

Cancer recurrence not only shortens the life span of cancer patients, but also leads to a decrease in QOL, so it needs to be controlled. Circulating tumor cells (CTCs), precursors of cancer recurrence, are present in circulating blood and are therefore targets for liquid biopsies. CTCs are divided into single CTCs and clustered CTCs. Metastatic foci are formed mainly from clustered CTC. Cluster CTC is characterized by long-term survival, activation of stem cell-related transcription factors, high tumorigenicity, and movability in capillaries in clusters, while changing its morphology. The clinical significance of CTC is as follows: diagnosis by cytology, monitoring of therapy, and molecular diagnostic samples. CTC extraction methods include size selection methods such as mesh, specific gravity method (CD45 cell elimination), and recovery using surface antigen as an index, rt-PCR, etc., which can be selected as appropriate [1]. The elucidation of the pathogenesis of CTC leads to the pathogenesis of cancer itself. Five research papers [2,3,4,5,6] and four review papers [7,8,9,10] related to CTC were published in the Special Issue “Circulating Tumor Cells: From the Laboratory to the Cancer Clinic”.

Representative CTC isolation methods include immunocapture methods, biophysical property positive selection methods, membrane filtration methods, and specific gravity methods. A membrane filtration method is suitable to observe the morphology of CTC which is a surrogate marker of tumorigenicity and potential as a stem cell. Sawabata et al. revealed that clustered CTCs, which are predictors of poor prognosis, were more frequently detected in cases of NSCLC with a pure solid appearance on CT than in tumors with mixed gland grass opacity (GGO) using a membrane filtration method [2]. This phenomenon explains the worse prognosis of patients with pure-solid tumors than those with mixed GGO tumors because the microenvironment of pure-sold tumors is different from that of mixed GGO tumors [11]. Staudte et al., using a specific gravity method, demonstrated intra-tumoral heterogeneity of PD–L1 expression in CTCs from head and neck cancer patients in addition to EGFR activation and the DNA damage repair in CTCs [3], which may represent a major constraint for further development of CTC assays, such as their use for the identification of therapeutic targets, the short–term evaluation of therapy response and early detection of disease progression.

Sample handling is also an important issue. Payne et al. demonstrated [4] the benefit of using a fixative blood collection tube to increase cell capture rate and preserve the CTC marker expression profile, which is a crucial observation when designing sample processing protocols for large cohort multicenter clinical trials investigating CTCs in any cancer type.

In addition to the above laboratory findings, the clinical importance of CTC is also reported. Sawabata et al. revealed that the clustered morphology is an indicator of poor prognosis in lung cancer patients [2]. Obermayr et al., shows that, given the heterogeneity and extreme scarcity of CTCs, a multifactorial analysis of CTCs is key, and that in addition to a long progression-free interval, the absence of CTCs after treatment was an independent predictor of an excellent outcome in patients who had already survived for five years. The absence of CTCs is also crucial in surgical cases. Adachi et al. reviewed [7] to discuss findings for the non-touch isolation technique in surgery, especially in surgery for non-small-cell lung cancer which aims to prevent the release of CTCs from the tumor nest to the bloodstream during surgery. Surgical manipulation has the potential to induce increments in intra- and postoperative CTCs. Non-touch isolation technique (NTIT), which is considered a pulmonary vein (PV)-first technique in surgery for Non-small cell lung cancer (NSCLC), theoretically prevents the release of these CTCs by early blockade of outflow vessels. However, evidence of the efficacy of NTIT in the prevention of CTC release and the post-operative prognosis is still unsatisfactory. This may be partially due to insufficient knowledge regarding the biological behavior of CTCs and the lack of technologies to detect CTCs. Immunotherapy is also a strategy of cancer therapy, that is reviewed by Maravelia et al. via liquid biopsy [8], including circulating tumor DNA (ctDNA), circulating RNA (e.g., microRNAs), circulating tumor cells (CTC) and extracellular vesicles (EVs) (e.g., exosomes) for disease monitoring in a non-invasive manner with discussion over the currently available technology that can enable the use of liquid biopsy as a diagnostic and prognostic tool, and the opportunities and challenges of the clinical application of liquid biopsy for immunotherapy of HCC.

CTCs are not the only ones playing an important role in liquid biopsy, so does cell-free DNA and so on. Shoukry et al. summarize the current applications of ctDNA in the diagnosis and surveillance of breast cancer [8]- liquid biopsies and ctDNA in particular are potentially bringing healthcare providers closer to that goal, and novel techniques such as BEAMing and next-generation sequencing have also made it easier to analyze breast cancer on a more personalized genomic level. D’yakonov shows [6] that the synthesized trienoic acids can claim the role of multitarget compounds that are able to simultaneously penetrate through various biological membranes, including mitochondrial, uncouple oxidation and phosphorylation, thereby reducing mitochondrial potential, as well as inhibiting topoisomerase I and affecting the main signaling pathways of cell proliferation. Kwiatkowska [10] reviewed to show how extensive and complex the role of tryptophan metabolism in modulating oncogenesis is and left the following comment. The world of science is facing an enormous challenge that must be resolved in order to understand the role of this metabolic pathway in cancer development. Despite the growing number of sources of information about individuals’ role and their involvement in cancer genesis, a lot of sources are still a puzzle, which provides an opportunity for further significant research. Overlapping immune mechanisms and a classic well-known process of tumorigenesis additionally emphasize a need for exploring the role of immunomodulators as tumor promotors or suppressors. Gaining this knowledge may change the face of oncological treatment in the future and improve patients’ survival and quality of life.

All of the articles in this Special Issue are thought-provoking and will lead to future research and clinical practice.

## References

[1] Pantel K., Brakenhoff R.H., Brandt B. (2008). Detection, clinical relevance and specific biological properties of disseminating tumour cells. Nat. Rev. Cancer.

[2] Sawabata N., Kawaguchi T., Watanabe T., Yohikawa D., Ouji-Sageshima N., Ito T. (2022). Pure Solid Pattern of Non-Small Cell Lung Cancer and Clustered Circulating Tumor Cells. Cancers.

[3] Staudte S., Klinghammer K., Jurmeister P.S., Jank P., Blohmer J.-U., Liebs S., Rhein P., Hauser A.E., Tinhofer I. (2022). Multiparametric Phenotyping of Circulating Tumor Cells for Analysis of Therapeutic Targets, Oncogenic Signaling Pathways and DNA Repair Markers. Cancers.

[4] Payne K., Brooks J.M., Taylor G.S., Batis N., Noyvert B., Pan Y., Nankivell P., Mehanna H. (2021). Immediate Sample Fixation Increases Circulating Tumour Cell (CTC) Capture and Preserves Phenotype in Head and Neck Squamous Cell Carcinoma: Towards a Standardised Approach to Microfluidic CTC Biomarker Discovery. Cancers.

[5] Obermayr E., Reiner A., Brandt B., Braicu E.I., Reinthaller A., Loverix L., Concin N., Woelber L., Mahner S., Sehouli J. (2021). The Long-Term Prognostic Significance of Circulating Tumor Cells in Ovarian Cancer—A Study of the OVCAD Consortium. Cancers.

[6] D’yakonov V.A., Makarov A.A., Dzhemileva L.U., Ramazanov I.R., Makarova E.K., Dzhemilev U.M. (2021). Natural Trienoic Acids as Anticancer Agents: First Stereoselective Synthesis, Cell Cycle Analysis, Induction of Apoptosis, Cell Signaling and Mitochondrial Targeting Studies. Cancers.

[7] Adachi H., Ito H., Sawabata N. (2022). Circulating Tumor Cells and the Non-Touch Isolation Technique in Surgery for Non-Small-Cell Lung Cancer. Cancers.

[8] Maravelia P., Silva D.N., Rovesti G., Chrobok M., Stål P., Lu Y.-C., Pasetto A. (2021). Liquid Biopsy in Hepatocellular Carcinoma: Opportunities and Challenges for Immunotherapy. Cancers.

[9] Shoukry M., Broccard S., Kaplan J., Gabriel E. (2021). The Emerging Role of Circulating Tumor DNA in the Management of Breast Cancer. Cancers.

[10] Kwiatkowska I., Hermanowicz J.M., Przybyszewska-Podstawka A., Pawlak D. (2021). Not Only Immune Escape—The Confusing Role of the TRP Metabolic Pathway in Carcinogenesis. Cancers.

[11] Katsumata S., Aokage K., Miyoshi T., Tane K., Nakamura H., Sugano M., Kojima M., Fujii S., Kuwata T., Ochiai A. (2018). Differences of tumor microenvironment between stage I lepidic-positive and lepidic-negative lung adenocarcinomas. J. Thorac. Cardiovasc. Surg..

